# Atrial septostomy and disease targeting therapy in pulmonary hypertension secondary to neurofibromatosis

**DOI:** 10.1186/s12890-016-0337-7

**Published:** 2016-12-07

**Authors:** George Giannakoulas, Panagiotis Savvoulidis, Vasilios Grosomanidis, Sophia-Anastasia Mouratoglou, Haralambos Karvounis, Stavros Hadjimiltiades

**Affiliations:** 1 Department of Cardiology, AHEPA University Hospital, Aristotle University of Thessaloniki, Stilp. Kiriakidi 1, Thessaloniki, 54637 Greece; 2 Department of Anesthesiology and Intensive Care Medicine, AHEPA University Hospital, Aristotle University of Thessaloniki, Stilp. Kiriakidi 1, Thessaloniki, 54637 Greece

**Keywords:** Neurofibromatosis, Pulmonary hypertension, Septostomy, Case report

## Abstract

**Background:**

Neurofibromatosis type 1 (NF1) is a rare multisystem genetic disorder. During the course of the disease it can be rarely complicated with pulmonary hypertension (PH) which confers a dismal prognosis.

**Case presentation:**

We describe the case of a 57-year-old female patient with NF1 complicated by severe precapillary PH despite dual disease-specific oral combination therapy. The patient was treated with initial atrial septostomy followed by administration of high-dose subcutaneous treprostinil with a favorable medium-term clinical and hemodynamic response.

**Conclusions:**

PH secondary to NF1 may be successfully treated with the combination of atrial septostomy and PH targeted therapy in selected patients.

## Background

Neurofibromatosis type 1 (NF1) is a genetic disease with a prevalence of 1:3500 in the general population, transmitted with the autosomal dominant inheritance pattern and with full penetrance characterized by prominent skin features (hyperpigmented macules, termed café-au-lait spots and nerve tumors), optical tumors and other central nervous system tumors, certain bony abnormalities, some learning deficits and an increased risk of certain non-nervous system cancers [1]. Mutations of the NF1 gene, which encodes neurofibromin and is located at chromosome 17q11.2, a negative regulator of the ras signal transduction pathway that has a role in both tumor suppression and regulation of cell growth and proliferation, are responsible for the NF1 [2].

A rare still morbid complication of NF1 is pulmonary hypertension (PH) which confers a dismal prognosis overall. To the best of our knowledge this is the first documented report of successful treatment of PH secondary to NF1 with atrial septostomy followed by escalation of PH targeted treatment.

## Case presentation

A 57-year-old female patient with a history of NF1 and PH initially diagnosed 3 years ago, already on conventional treatment with supplemental oxygen and anticoagulation, as well as disease-specific double oral combination therapy (ambrisentan 10 mg od and tadalafil 40 mg od) was admitted to the hospital because of worsening dyspnea on mild exertion and a presyncopal episode.

Her past medical history was notable for surgically repaired pyloric stenosis and 2 abortions during the third trimester of gestation due to intrauterine fetal death, followed by a successful pregnancy. The patient had already undergone an extensive workup which excluded known causes of PH. Lung ventilation/perfusion scan was not suggestive for chronic thromboembolic disease and high-resolution CT of the lungs was negative for interstitial lung disease. Blood tests excluded other causes of pulmonary hypertension.

**Fig. 1:**
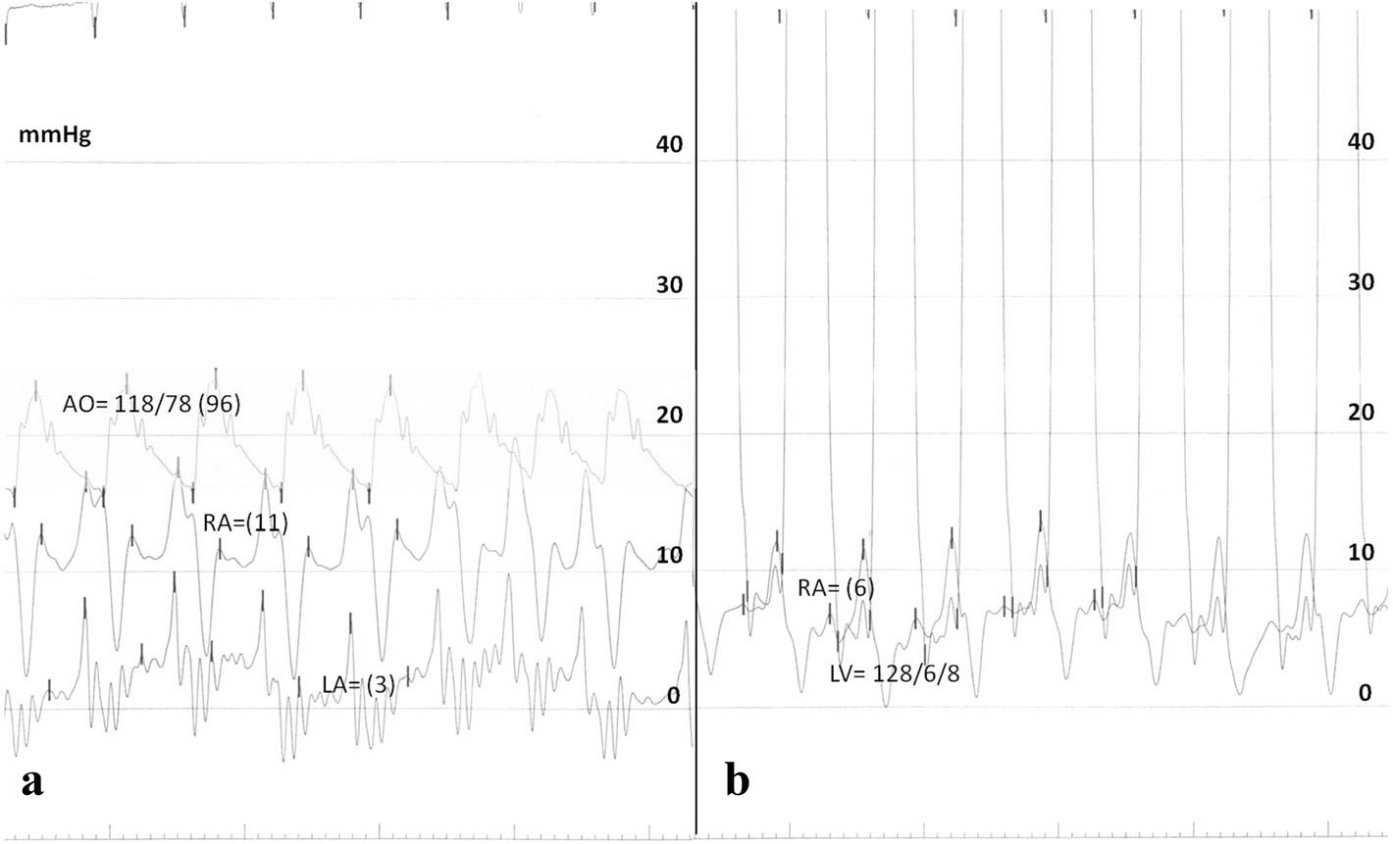
**a** Simultaneous recording of *right atrial* (RA) and *left atrial* (LA) pressures, before the creation of the balloon atrial septostomy. **b** Simultaneous recording of *right atrial* (RA) and *left ventricular* (LV) pressures, demonstrating the fall in the mean RA and the increase in the LV diastolic pressures as compared to the LA pressure in A. The aortic pressure (AO) is recorded on a pressure scale of 0–200 mmHg. Mean pressures are in parentheses

Considering the patient’s clinical deterioration, the decision was made to proceed immediately with atrial septostomy, due to inherent difficulties in the reimbursement of prostanoids in our hospital at that time. The patient had an uneventful procedure with graded balloon dilation of the interatrial septum with gradually increasing inflated balloons diameter of 6, 8 and 10 mm. A moderate decrease in right atrial pressure (Fig. 1), a mild increase in cardiac index and, as expected, a decrease in SaO2 was noted (Table 1) due to the right-to-left shunting through the atrial septum. The patient remained clinically and hemodynamically stable and was discharged a few days later.

**Table 1:** Serial hemodynamic, functional and neurohormonal measurements

Parameter	Jan 2011	PreBAS Mar 2014	PostBAS	Trepostinil Mar 2015	Trepostinil Feb 2016
HR (beats/min)	76	91	90	71	70
RAP (mmHg)	(3)	(11)	(7)	(4)	(6)
PAP (mmHg)	68/26 (43)	130/57 (84)	135/56 (84)	106/36 (63)	77/30 (49)
PAWP/LAP* (mmHg)	(6)	(2)*	(7)*	(7)	(9)*
Ao (mmHg)	130/68 (93)	122/65 (84)	124/65 (84)	119/71 (87)	128/65 (85)
PVR (WU)	8.3	27.1	26.3	11.6	6.8
CI (l/min/m^2^)	2.9	1.9	2.0	3.1	3.7
SaO2 (%)	97 at rest 90 (during exercise)	98	93	95 at rest (70 during exercise)	98 at rest (75 during exercise)
6MWT (m)	489	230^a^	335^b^	345	399
NT-proBNP (pg/ml)	125	3402^a^	1507^b^	NA	161

Mean pressures are shown in parentheses

*BAS*: balloon atrial septostomy, *HR*: heart rate, *RAP*: Right atrial pressure, *PAP*: Pulmonary artery pressure, *LAP*: Left atrial pressure, *PCWP*: Pulmonary capillary wedge pressure, *Ao*: Aortic pressure, *PVR*: Pulmonary vascular resistance, *CI*: Cardiac index, *WU*: Wood Units, *6MWT*: 6-min walking test, *NT-proBNP*: N-terminal pro-brain natriuretic peptide, *NA*: not available

*Asterisk indicates that the pressure was measured in the left atrium

^a^2 weeks prior to the procedure

^b^1 month after the procedure

After an initial modest improvement in symptoms, six-minute walk test (6MWT) and NT-proBNP at 2 months, access to prostanoids was eventually gained and the patient was started on subcutaneous treprostinil with gradual increase in the administered dose up to 100 ng/kg/min in a time period of 7 months.

**Fig. 2:**
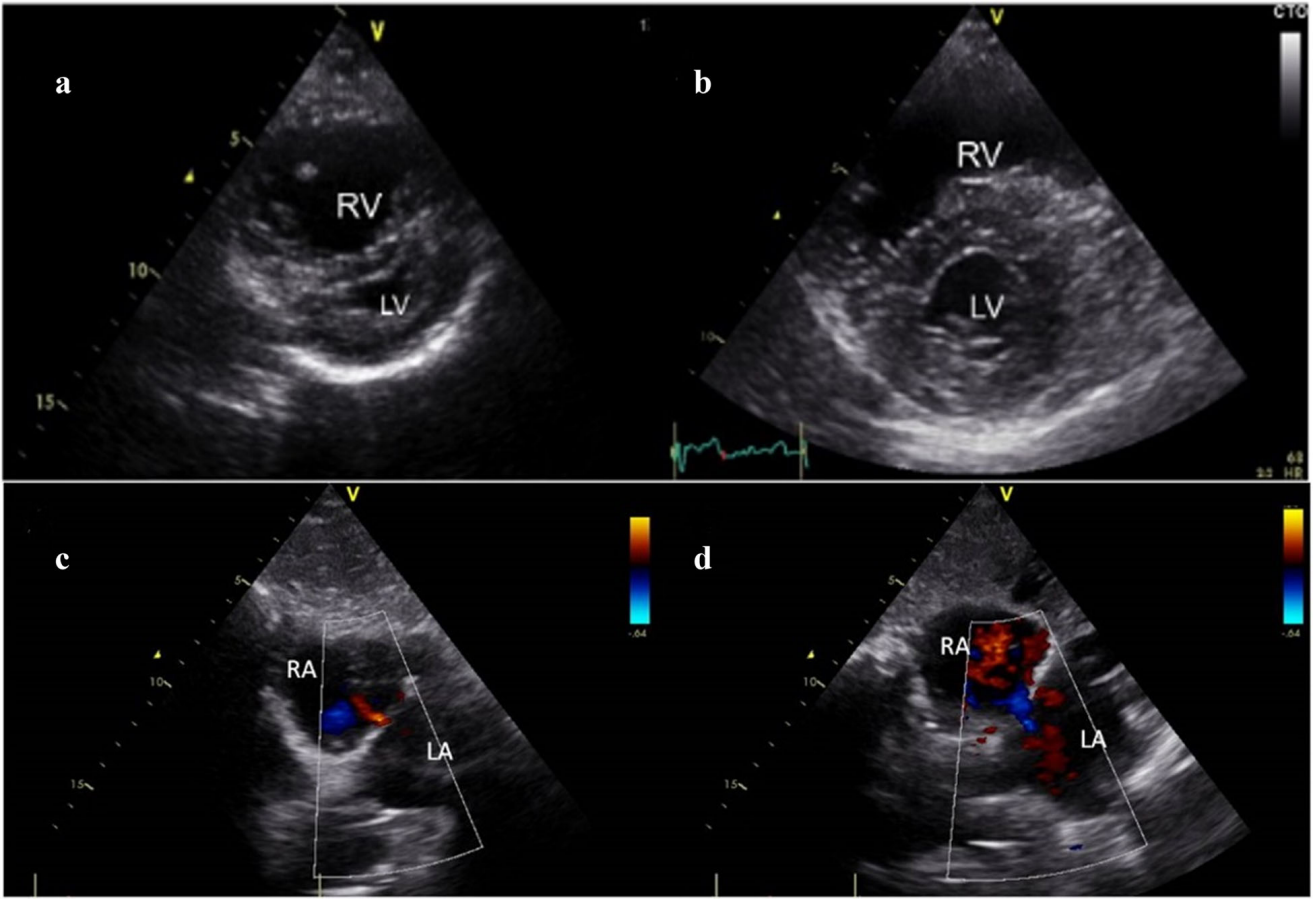
**a**, **b** Short-axis transthoracic echocardiographic view demonstrating the positive remodelling of the *right ventricle* (RV) 2 years after the atrial septostomy and the initiation of subcutaneous treprostinil. LV = *left ventricle* **c** Subxiphoid echocardiographic view of the persistent flow from the *left atrium* (LA) to the *right atrium* (RA) at rest **d** Reversal of flow from RA to LA after isometric exercise

The patient during a follow-up period of 2 years demonstrated gradual improvement and at present remains in World Health Organization class 2 with significant hemodynamic improvement (Table 1). A recent transthoracic echocardiogram demonstrated the positive remodeling of the right ventricle (Fig. 2a, b) and the persistence of the atrial septostomy (Fig. 2c). The shunt flow was small and at rest was from the left atrium to the right atrium but after 2 min of isometric exercise (hand grip and feet in cycling position) the shunt became right-to-left (Fig. 2d).

## Discussion

A few cases suggesting an association of NF1 gene mutation and precapillary PH have been published [3, 4]. Plexiform lesions similar to those observed in human idiopathic PAH have been described, implying that vasculopathy of neurofibromatosis might underlie the pathophysiology of PH, a severe complication, which seems to show a predilection for women with severe clinical and hemodynamic impairment and a poor outcome [5].

Herein, we report a case of a patient with NF1 and severe precapillary PH, without parenchymal lung disease, yet with prominent pulmonary vascular involvement, treated with atrial septostomy, followed by the administration of subcutaneous treprostinil. The medium-term clinical response after 2 years was impressive with improvement in her functional class, 6MWT distance, hemodynamic parameters and NT-proBNP. To the best of our knowledge this is the first published case of PH secondary to neurofibromatosis treated successfully with the combination of atrial septostomy and aggressive PH targeted therapy.

The favorable clinical response of our patient to the atrial septostomy and the further gradual, but more pronounced, improvement with the chronic administration of subcutaneous treprostinil suggests that pulmonary vasculopathy is the underlying mechanism of PH in these patients. The creation of a shunt that decreases the preload of the right ventricle and increases that of the left ventricle with concomitant increase in the cardiac output makes it easier to initiate and uptitrate the prostanoid infusion. The long-term effect of the persistence of the shunt in our case, following the decrease in the right sided pressures on chronic treprostinil therapy, is not known; whether the reversal of the shunt during exercise by decreasing the load on the right ventricle will continue to be beneficial, despite the arterial oxygen desaturation, is not clear [6].

There are very few cases in the literature with the utilization of PAH-targeted therapies in these patients [7–9]. Our patient improved significantly after the initiation of treprostinil, while double oral therapy had initially failed. The development of PH in patients with NF1 confers a dismal prognosis and therefore, the earlier and aggressive treatment with prostanoids should be considered. Finally, listing these patients for lung transplantation is a matter of debate, since a higher than the general population incidence of malignancies has been described [10].

## Conclusions

This report demonstrates the successful management of a patient with PH secondary to NF1 with atrial septostomy in conjunction with PAH targeted therapy. Looking ahead, national and international prospective registries will shed light on the pathophysiology, and especially optimal therapy and prognosis of neurofibromatosis-associated PH especially in the modern era of targeted PAH treatment.

## Abbreviations

6MWT: Six minute walk test
NF1: Neurofibromatosis type I
NT-proBNP: N-terminal pro-brain natriuretic peptide
PH: Pulmonary hypertension
PAH: Pulmonary arterial hypertension
